# Basal Ganglia Germ Cell Tumors With or Without Sellar Involvement: A Long-Term Follow-Up in a Single Medical Center and a Systematic Literature Review

**DOI:** 10.3389/fendo.2021.763609

**Published:** 2021-11-10

**Authors:** Yi Zhang, Li Wang, Wenbin Ma, Hui Pan, Renzhi Wang, Huijuan Zhu, Yong Yao

**Affiliations:** ^1^Department of Neurosurgery, Peking Union Medical College Hospital, Chinese Academy of Medical Science and Peking Union Medical College, Beijing, China; ^2^Department of Endocrinology, Peking Union Medical College Hospital, Chinese Academy of Medical Science and Peking Union Medical College, Beijing, China

**Keywords:** basal ganglia germ cell tumors, sellar involvement, delayed diagnosis, independent prognostic risk factors, surgical therapy

## Abstract

**Background:**

Basal ganglia germ cell tumors (BGGCTs) represent an extremely rare subset of tumors about which little is known. Some patients suffer from tumor dissemination, such as sellar involvement. This study aimed to evaluate the independent prognostic risk factors of patients with BGGCTs with or without sellar involvement.

**Methods:**

Sixteen patients were diagnosed with BGGCTs at Peking Union Medical College Hospital from January 2000 to December 2020. A literature review was performed on the online databases Medline and PubMed, and 76 cases in the 19 retrieved articles were identified at the same time. The data regarding biochemical tests, radiological examinations, and outcomes during follow-up were analyzed.

**Results:**

Of 92 patients in this study, seven patients were clinically diagnosed as germinomas, with the remaining 85 patients receiving surgery. Fifty-two patients suffered from multifocal lesions or tumor dissemination. The patients with BGGCTs demonstrated a significant male predilection. The patients with delayed diagnosis more likely had cognitive disturbance (p = 0.028), mental disturbance (p = 0.047), and diabetes insipidus (p = 0.02). Multivariate analysis demonstrated that the independent poor prognostic risk factors of patients with BGGCTs were delayed diagnosis [odd ratio (OR) 2.33; 95% CI 1.02–5.31], focal radiotherapy (OR 4.00; 95% CI 1.69–9.49), and non-pure germinoma (OR 4.64; 95% CI 1.76–12.22).

**Conclusions:**

The delayed diagnosis, focal radiotherapy, and non-pure germinoma were associated with a poorer prognosis for patients with BGGCTs with or without sellar involvement.

## Introduction

Intracranial germ cell tumors (GCTs) are rare, heterogeneous, and management challenging, with a geographically variable incidence of 0.6–2.7 per million (1, 2). GCTs usually develop in the region of the third ventricle along the midline axis, including sellar region, pineal region, or both regions involved (3, 4). In rare circumstances, GCTs also originate off the midline in the basal ganglionic region, thalamus, or other ventricular sites. It is reported that GCTs in basal ganglionic region are more likely to be germinomas, which are prone to delayed diagnosis for their insidious onset and atypical clinical presentation (3, 5, 6).

Basal ganglia consist of subcortical nuclei embedded in the deep brain hemispheres responsible for control of movement, behavior, cognition, and emotions (7). Slowly progressive hemiparesis and cognitive decline are frequent in patients with GCTs in the basal ganglia (8, 9). In this study, we define basal ganglia area based on the imaging, including internal capsule and peripheral white matter in addition to anatomical BG (striatum, claustrum, and amygdaloid body).

Due to the rarity of basal ganglia germ cell tumors (BGGCTs), there is a paucity of research on it and a lack of study on large populations. Our study aims to determine the demographic and clinical characteristics, associated factors, and impact of delayed diagnosis and treatment on outcome of BGGCTs with or without sellar involvement. We reviewed 92 BGGCT patients with complete clinical information and follow-up data since 2000, including 16 patients diagnosed with BGGCTs at our institution and 76 cases from other published studies. To the best of our knowledge, this study included the largest population so far.

## Materials and Methods

### Patient Selection

Between January 2000 and December 2020, 16 patients were diagnosed with BGGCTs at Peking Union Medical College Hospital (PUMCH). Medical information was collected, including patients’ demographics, symptoms and physical examination, clinical course, endocrine tests, radiological examinations, treatment modalities, and outcomes during follow-up. All patients underwent magnetic resonance imaging (MRI), including T1-weighted imaging (T1WI), T2-weighted imaging (T2WI), and contrast-enhancement T1WI (CE-T1WI). The tumor size, locations, calcification, cyst, hemorrhage, parenchymal atrophy, and hydrocephalus were recorded. The alpha-fetoprotein (AFP) (normal value range at PUMCH, 0–20 ng/ml) and β-human chorionic gonadotropin (β-HCG) (normal value range at PUMCH, 0–5 IU/L) in serum level were examined routinely.

### Literature Review

The online database Medline and PubMed were searched for the phrases “basal ganglia” and “germ cell tumors” from January 2000 to December 2020. Forty results were totally returned. Furthermore, 11 articles were obtained by reviewing citations in the retrieved data. Cases were excluded if (1) articles were not published in English (2), case reports overlapped in two or more papers (3), individual basic data were not included in the articles, and (4) the follow-up time was less than 1 year. Finally, 76 cases in the 19 retrieved articles were identified.

### Definition

Tumor size was recorded as the measured maximum diameter in MRI scans. Delayed diagnosis was defined as an interval of more than 6 months from the onset of symptoms to the date of diagnosis. Overall survival (OS) was defined as the length of time from the date of initial treatment to that of death or last follow-up. Progression-free survival (PFS) was defined as the length of time during and after the treatment that a patient lives without progression of disease. Odds ratio (OR) is a measure of association between an exposure and an outcome, representing the odds that an outcome will occur given a particular exposure, compared to the odds of the outcome occurring in the absence of that exposure. Confidence interval (CI) is a type of estimate computed from the observed data, giving a range of values for an unknown parameter.

### Statistical Analysis

Quantitative data were described as mean ± SD or median (25 and 75th percentiles). Pearson chi-square test, Continuity correction chi-square test, or Fisher’s exact test was used to assess the statistical significance in 2 × 2 table. The significant difference of R × C cross-table was evaluated by Wilcoxon rank test, Kruskal–Wallis test, or Spearman rank correlation. The survival curve was generated by Kaplan–Meier method. Multivariate logistic regression was used to identify the risk factors. The data were analyzed using SPSS 13.0 (IBM, NY, USA) and GraphPad Prism 6 (GraphPad Software, CA, USA).

## Results

### Demographics

**Figure 1:**
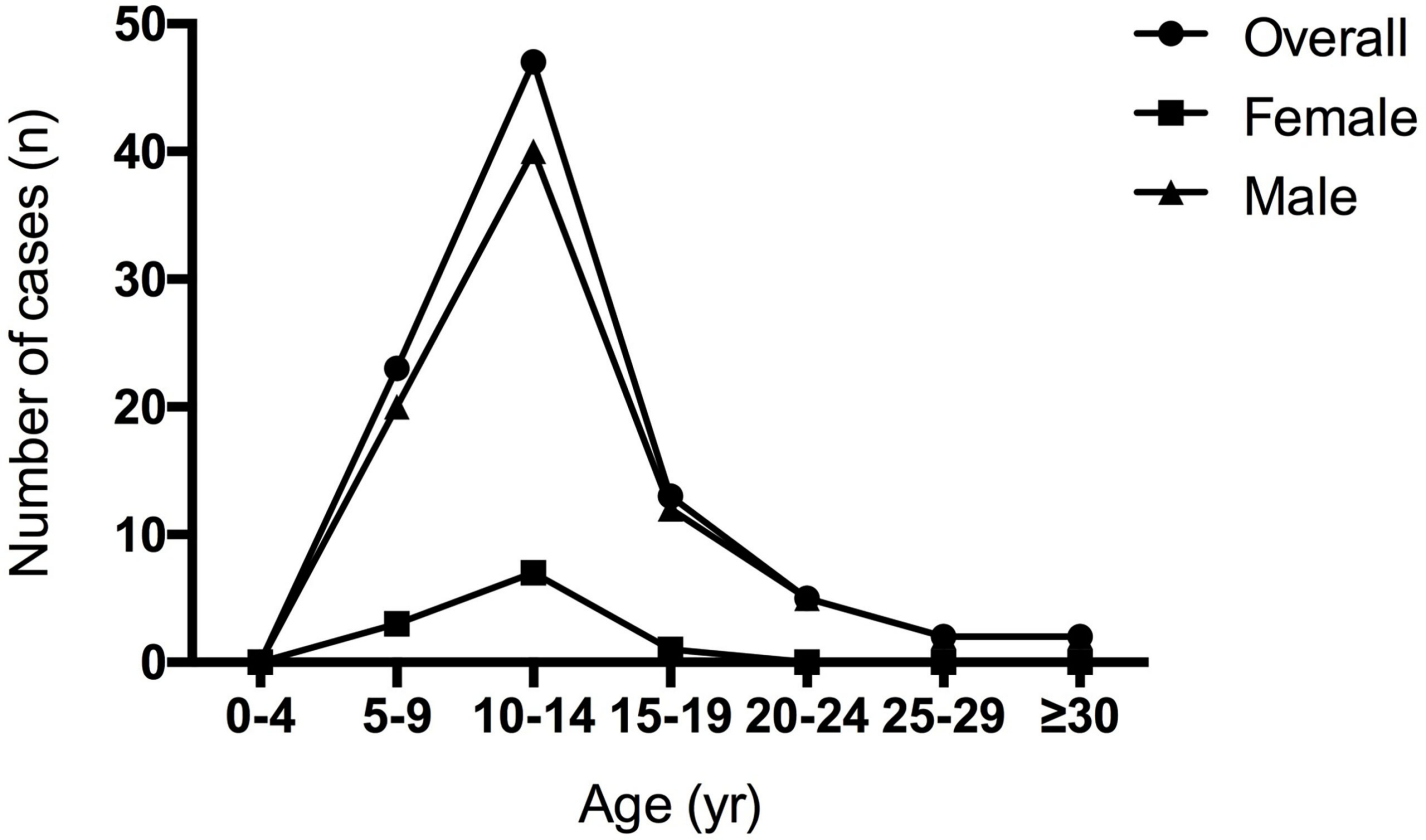
Age of 92 patients with basal germ cell tumors.

A total of 92 cases were diagnosed with BGGCTs, including 16 cases from our medical center. The male/female was 7.36:1, demonstrating a significant male predilection of BGGCTs. The median age at diagnosis was 12.5 years (interquartile range, 9.75–14 years). The age data showed that patients with BGGCTs tended to be diagnosed in the 5–15 years (**Figure 1**).

### Clinical Features

The clinical features of patients with BGGCTs are shown in **Table 1**. The most frequent symptom was hemiparesis (67%), followed by cognitive disturbance (24.2%), nausea/vomiting (22%), and headache (16.5%). In addition, 18 patients suffered from diabetes insipidus. The other neurologic signs included dystonia (15.4%), positive pathologic reflex (15.4%), and gait abnormality (15.4%). The median duration of symptoms was 7 months (interquartile range, 1.5–23.1 months).

**Table 1:** Clinical features of patients with germ cell tumors with or without sellar involvement.

Presenting Symptom or Sign	Overall (n = 92)		Delayed (n = 53)		Undelayed (n = 39)		X^2^	p
Hemiparesis	61	67.0%	35	67.3%	26	66.7%	0.004	0.949
Dystonia	14	15.4%	8	15.4%	6	15.4%	0	1
Positive pathologic reflex	14	15.4%	8	15.4%	6	15.4%	0	1
Gait abnormality	14	15.4%	10	19.2%	4	10.3%	1.379	0.24
Cognitive disturbance	22	24.2%	17	32.7%	5	12.8%	4.801	0.028^#^
Paresthesia	6	6.6%	5	9.6%	1	2.6%	0.836	0.36
Syncope	3	3.3%	2	3.8%	1	2.6%	0	1
Seizure	6	6.6%	4	7.7%	2	5.1%	0.04	0.951
Impulsive behavior/fluctuating emotional control	9	9.9%	6	11.5%	3	7.7%	0.064	0.8
Mental disturbance	8	8.7%	8	15.1%	0	0.0%	3.95	0.047^#^
Headache	15	16.5%	6	11.5%	9	23.1%	2.155	0.142
Nausea/vomiting	20	22.0%	10	19.2%	10	25.6%	0.534	0.465
Visual changes	7	7.7%	5	9.6%	2	5.1%	0.158	0.691
Slow growth	6	6.6%	4	7.7%	2	5.1%	0.004	0.951
Menstrual changes	2	2.2%	2	3.8%	0	0.0%	0.266	0.606
Decreased libido	3	3.3%	3	5.8%	0	0.0%	0.869	0.351
Precocious puberty	10	11.0%	7	13.5%	3	7.7%	0.283	0.595
Dry skin and mucosa	6	6.6%	6	11.5%	0	0.0%	3.126	0.077
Anorexia	5	5.5%	4	7.7%	1	2.6%	0.357	0.55
Fatigue	8	8.8%	7	13.5%	1	2.6%	2.081	0.149
Diabetes insipidus	18	19.6%	15	28.3%	3	7.7%	5.425	0.02^#^
Consciousness disturbance	3	3.3%	1	1.9%	2	5.1%	–	1
Pigmentation	4	4.4%	3	5.8%	1	2.6%	0.049	0.825

^#^p < 0.05.

### Tumor Markers

All patients received the alpha-fetoprotein (AFP) and β-human chorionic gonadotropin (β-HCG) test in serum. Elevated β-HCG was observed in 29 patients, with elevated AFP occurring in seven patients. Among them, three patients were identified with both increased β-HCG and increased AFP levels.

### Radiologic Features

All patients underwent at least one cranial MRI. The GCT mass was only in basal ganglia in 40 patients. And 52 patients suffered from multifocal lesions or tumor dissemination. Ten patients had only a subtle patchy lesion visible mainly in T2WI. The maximum of tumor size was 65 mm. On MRI, the tumors in 61 patients (66.3%) demonstrated hypo- to iso-intensity on T1-wighted sequences, with 89 patients (96.7%) showing hyper-intensity on T2-wighted sequences. The most typical enhancement patterns were heterogeneous (81.5%) after administration of intravenous contrast material. The presence of cystic formation was observed in 30 cases (32.6%), with calcification in 18 cases (19.6%), obstructive hydrocephalus in 11 cases (12.0%), and intratumor hemorrhage in 11 cases (12.0%), respectively. Notably, parenchymal atrophy was identified in 42 cases (46.7%), which mainly presented with cerebral peduncle.

### Delayed diagnosis

Fifty-three of 92 patients with BGGCTs had a clinical course longer than 6 months. The clinical manifestation of patients with BGGCTs with or without delayed diagnosis is shown in **Table 1**. The three most common symptoms in patients with delayed diagnosis were hemiparesis (67.3%), cognitive disturbance (32.7%), and diabetes insipidus (28.35). The patients with delayed diagnosis more likely had cognitive disturbance (p = 0.028), mental disturbance (p = 0.047) and diabetes insipidus (p = 0.02).

The characteristics of patients with BGGCTs with delayed diagnosis are shown in **Table 2**. The male/female ratio of patients with delayed diagnosis is 9.6:1. The patients with delayed diagnosis more likely had multifocal lesions/tumor dissemination (p = 0.003). There were no statistically significant differences in tumor size and histological type (p > 0.05).

**Table 2:** Characteristics of BGGCT patients with and without a delay in diagnosis.

	Delayed diagnosis (n = 53)	Undelayed diagnosis (n = 39)	p-value
Age (Mean ± SD)	13.8 ± 6.0	12.4 ± 3.2	
Sex (n)			0.78
Male	48	33	
Female	5	6	
Tumor size (mm)			0.38
≥30	25	22	
<30	28	17	
Tumor location (n)			**0.003^#^**
Solitary lesion	16	24	
Multifocal lesions/ Tumor dissemination	37	15	
Histological type(n)			0.57
Germinoma	47	33	
NGGCT	6	6	

Data were presented as mean ± SD or N.

p-values in bold font indicate statistical significance. ^#^p < 0.01.

NGGCT, non-germinomatous germ-cell tumors; GCTs, germ cell tumors; BGGCTs, basal ganglia germ cell tumors.

### Diagnosis and Treatment

Seven patients with elevated β-HCG and normal AFP were clinically diagnosed as germinomas. The remaining 85 patients received surgery, including biopsy in 63 patients and debulking surgery in 22 patients. Twelve patients were pathologically diagnosed as non-germinomatous germ cell tumors (NGGCTs), with 65 patients as germinomas pathologically. Histological examination confirmed choriocarcinoma in two patients, teratoma in one patient, yolk sac tumor in one patient, and mixed GCT in eight patients.

Fifty-five patients received a combination of chemotherapy (CT) and radiotherapy (RT), while 21 patients received RT alone and 10 patients received CT alone. The irradiation fields were craniospinal irradiation (CSI) in 37, whole-brain irradiation (WBI) in 17, whole-ventricle irradiation (WVI) in 12, and only primary boost (PB) in 10. The CT protocols included cisplatin–etoposide (PE), carboplatin–etoposide (CE), ifosfamide–cisplatin–etoposide (ICE), bleomycin–etoposide–cisplatin (BEP), cisplatin–vincristine–bleomycin (PVB), and so on.

### Clinical Outcomes

**Figure 2:**
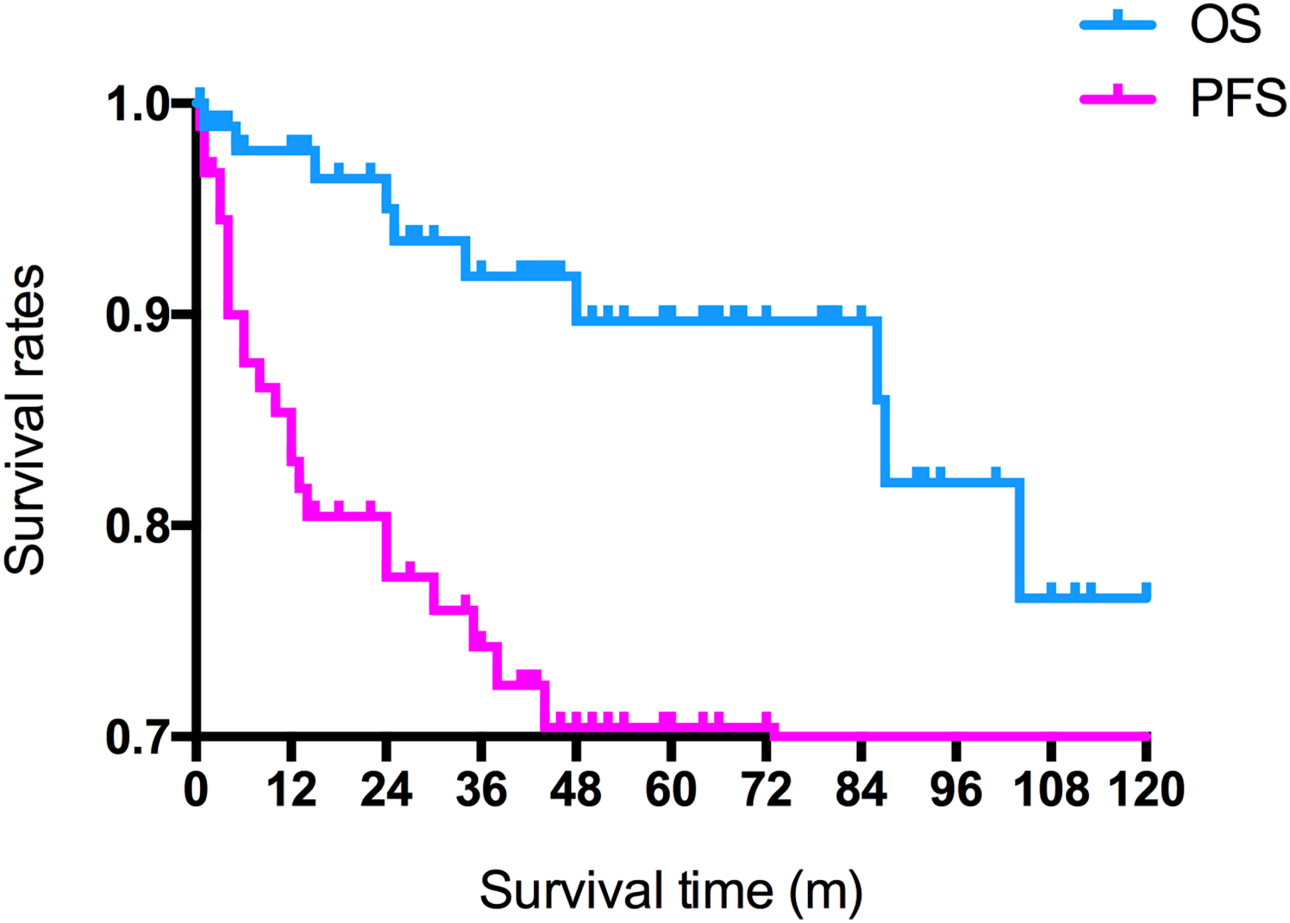
Overall and progression-free survival rates of all patients. OS, overall survival; PFS, progression-free survival.

**Figure 3:**
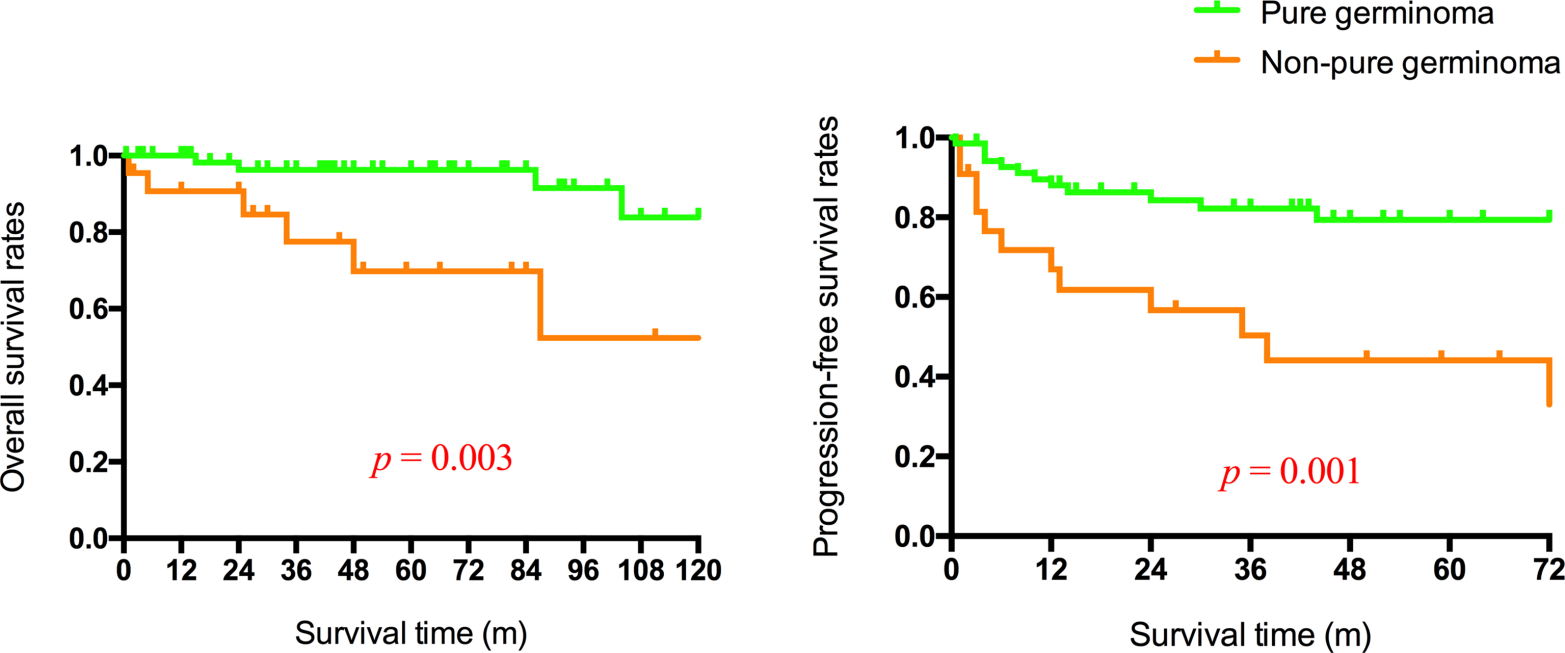
Overall and progression-free survival rates of patients with germinoma or non-pure germinoma.

All patients underwent follow-up without loss (**Table 3**). The follow-up time ranged from 0.5 to 205 months. At the time of the latest follow-up, 38 patients showed complete remission, 17 patients showed partial remission, 12 patients were alive with tumor size stable after the completion of all planned treatments, and 25 patients had progressive disease or were dead. The 1-, 3-, and 5-year OS rate was 97.9%, 93.0%, and 87.5%, respectively. The 1-, 3-, and 5-year PFS rate was 90.2%, 83.0%, and 83.0%, respectively (**Figure 2**). Considering the histological type, the mean survival time and PFS time of patients with pure germinoma were much longer than the ones with non-pure germinomas, 59.64 ± 5.05 months vs. 40.91 ± 10.78 months (p = 0.003) and 52.77 ± 5.17 months vs. 38.41 ± 11.74 months (p = 0.001), respectively (**Figure 3**).

**Table 3:** Univariate analysis of risk factors of patients with BGGCT.

	CR (n = 38)	PR (n = 17)	NC (n = 12)	PD (n = 25)	p-value
Sex (n)					0.630
Male	34	16	9	22	
Female	4	1	3	3	
Age at presentation (n)					0.567
<18	33	15	11	20	
≥18	5	2	1	5	
Delayed diagnosis (n)					0.017^*^
Yes	17	10	7	19	
No	21	7	5	6	
Cognitive disturbance (n)					0.245
Yes	6	6	3	7	
No	32	11	9	18	
Diabetes insipidus (n)					0.356
Yes	4	6	4	4	
No	34	11	8	21	
Tumor size (n)					0.675
≥30 mm	18	7	11	11	
<30 mm	20	10	1	14	
Tumor location (n)					0.366
Solitary	20	5	4	11	
Multifocal/Disseminated	18	12	8	14	
Operation types^a^ (n)					0.628
Debulking	11	3	2	6	
Biopsy	23	13	10	17	
None	4	1	0	2	
Combined CT and RT (n)					0.481
Yes	22	13	9	11	
No	16	4	3	14	
RT field (n)					0.001^#^
CSI or WVI or WBI	32	10	11	8	
Focal PB or others^b^	6	7	1	17	
Histological type (n)					0.002^#^
Pure germinoma	34	14	9	13	
Others^c^	4	3	3	12	

^#^p < 0.01.

^*^p < 0.05.

Operation types^a^ were calculated by Kruskal–Wallis test.

Others^b^ including non-radiotherapy and other types.

Others^c^ including germinoma with STGC and NGGCT.

NGGCT, non-germinomatous germ-cell tumor; GCT, germ cell tumor; STGC, syncytiotrophoblastic giant cell; CR, complete remission; PR, partial remission; NC, no change; PD, progressive disease; CSI, craniospinal irradiation; WBI, whole-brain irradiation; WVI, whole-ventricle irradiation; PB, primary boost; CT, chemotherapy; RT, radiotherapy; BGGCTs, basal ganglia germ cell tumors.

**Figure 4:**
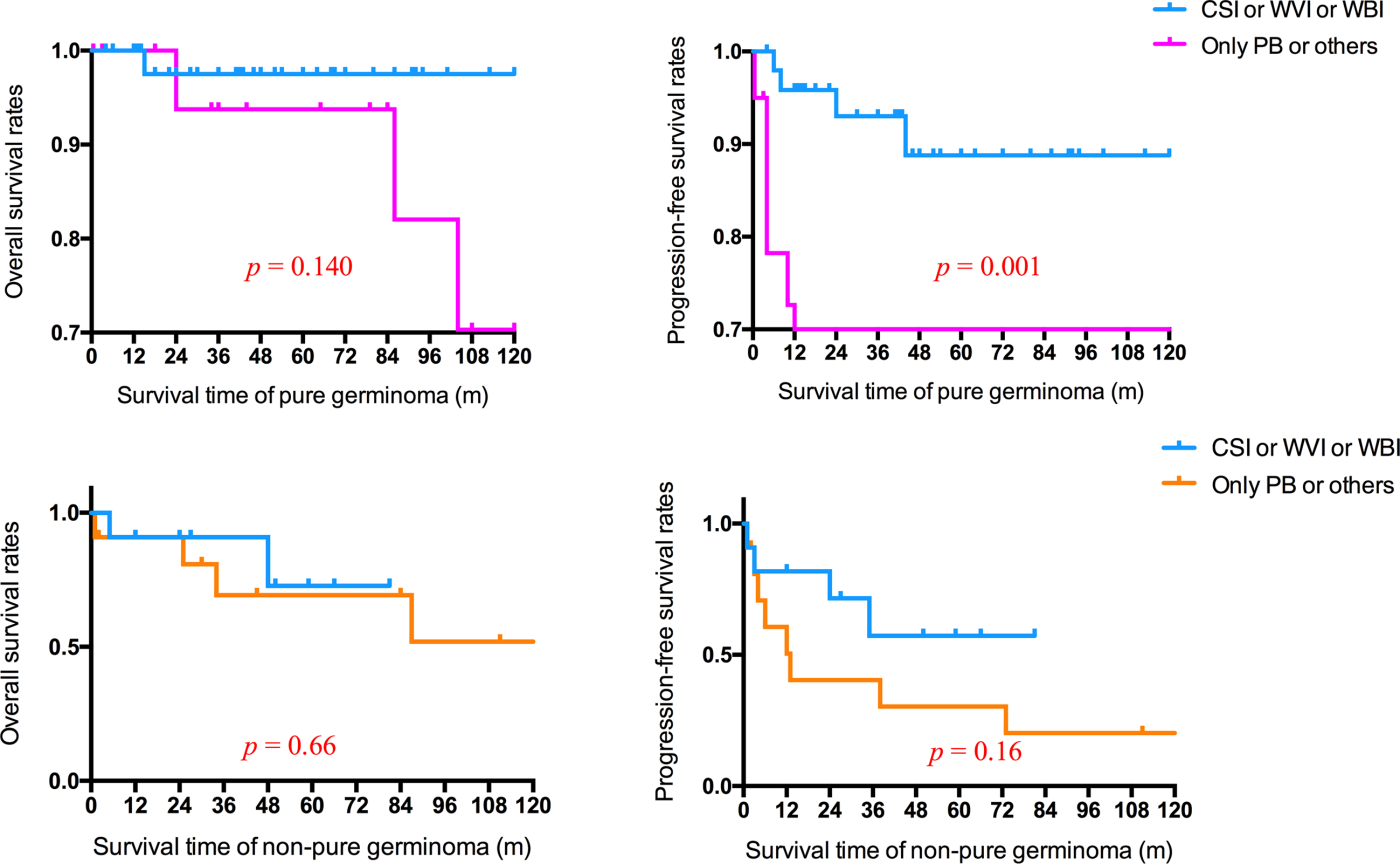
Overall and progression-free survival rates of patients with pure germinoma or non-pure germinoma in different radiotherapy fields. CSI, craniospinal irradiation; WVI, whole-ventricle irradiation; WBI, whole-brain irradiation; PB, primary boost.

For patients with non-pure germinoma, there were no statistically significant differences in the mean survival time between non-focal RT and focal RT (p = 0.66), neither did mean PFS time (p = 0.16). However, for patients with pure germinoma, non-focal RT was associated with a better PFS time than focal RT (p = 0.001) (**Figure 4**).

**Figure 5:**
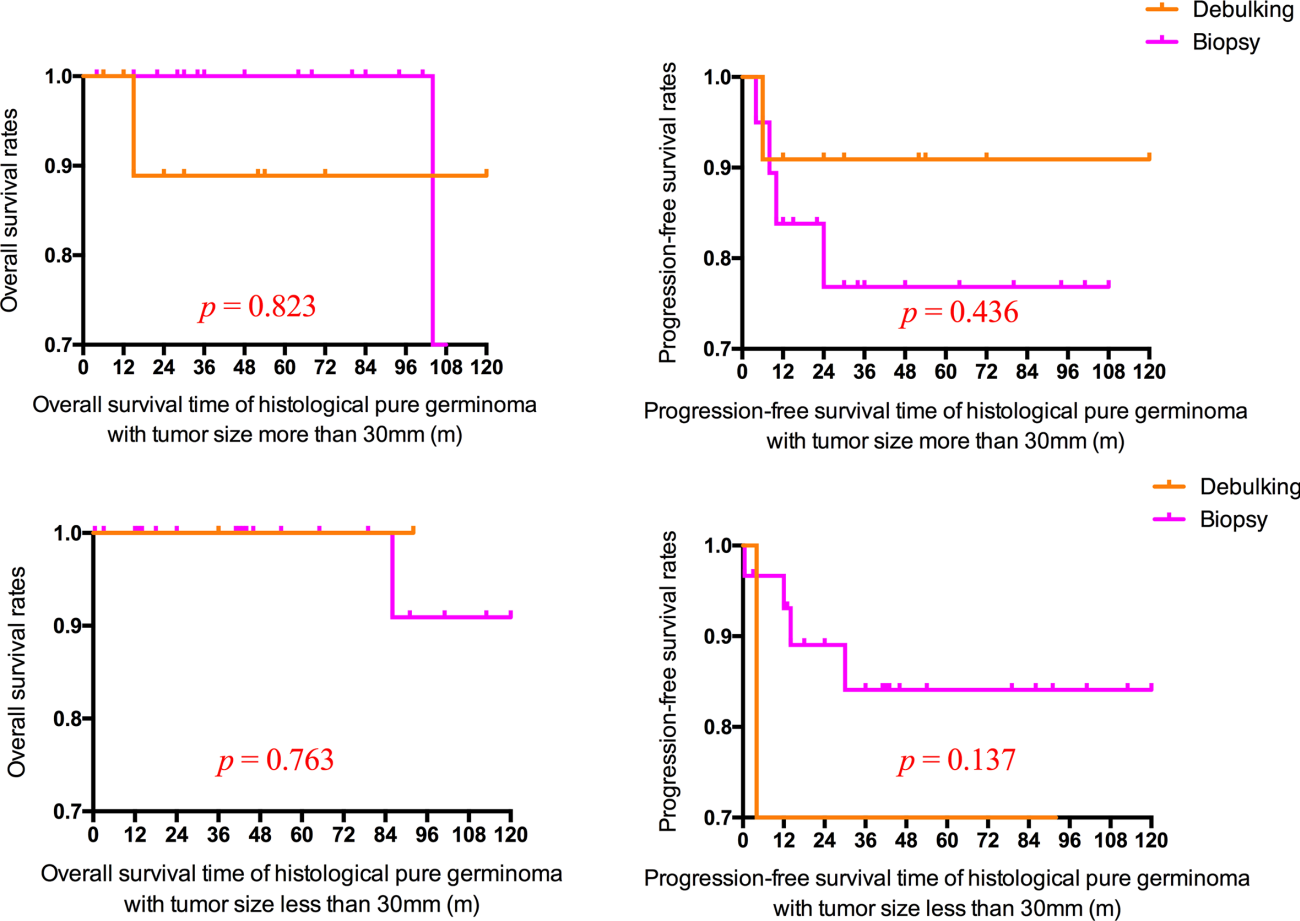
Overall and progression-free survival rates of patients with histologically confirmed pure germinoma with different tumor sizes in different operation types.

Given the tumor size and histological type over surgical resection, there were no statistically significant differences in mean OS and PFS time between patients with debulking and biopsy (p > 0.05) (**Figure 5**).

### Prognostic Factors

Some demographic features, clinical manifestations, histological features, radiological features, and different treatment protocols were used for risk stratification (**Table 3**). The diagnosis time (with delayed diagnosis vs. without delayed diagnosis, p = 0.017), the RT field (non-focal RT vs. focal RT, p = 0.001), and histological type (pure germinoma vs. non-pure germinoma, p = 0.002) were significantly related to survival. Sex, presentation time, cognitive disturbance, diabetes insipidus, tumor size, tumor location, operation type, and combination of RT and CT were not significantly related to survival (p > 0.05).

The previous three statistically significant factors were analyzed by multivariate logistic regression analysis (**Table 4**). On the premise that regression model had good-fitness and statistical significance, the diagnosis time, RT field, and histological type were the independent risk factors associated with BGGCT prognosis. Patients with delayed diagnosis (OR 2.33; 95% CI 1.02–5.31), focal RT (OR 4.00; 95% CI 1.69–9.49), and non-pure germinoma (OR 4.64; 95% CI 1.76–12.22) had worse prognosis.

**Table 4:** Multivariate logistic regression of parameters associated with prognosis of BGGCTs.

Variable	OR	95% CI	p-value
Delayed diagnosis	2.33	1.02–5.31	0.045^*^
RT field	4.00	1.69–9.49	**0.002^#^**
Histological type	4.64	1.76–12.22	**0.002^#^**

Results were obtained in ordinal logistic regressions, adjusting for sex (male vs. female), age (≥18 vs. <18), cognitive disturbance (yes vs. no), diabetes insipidus (yes vs. no), tumor size (≥30 mm vs. <30 mm), tumor location (solitary vs. multifocal/disseminated), operation type (debulking vs. biopsy), and RT and CT (yes vs. no).

p-values in bold font indicate statistical significance. ^#^p < 0.01, ^*^p < 0.05.

OR, odds ratio; CI, confidence interval; CT, chemotherapy; RT, radiotherapy; GCT, germ cell tumor; BGGCTs, basal ganglia germ cell tumors.

## Discussion

The interquartile range of age at diagnosis is from 9.75 to 14 years, with a median age of 12.5 years and a peak between 10 and 14 years. As shown in **Figure 1**, it is noticeable that males are more likely to have BGGCTs with or without sellar involvement in all age groups. Young-age onset and male predominance are two remarkable features of BGGCTs, which is consistent with previous studies (6, 10).

Basal ganglia are a group of evolutionarily conserved deep forebrain nuclei, which form multiple parallel loops and reentering circuits and are involved in motor, cognition, and affective control (7, 11). Abnormalities in the basal ganglia area can lead to movement disorders, alterations of mood, and cognitive disorders. Distinctive clinical features can lead to early diagnosis and optimize the treatment. However, patients with BGGCTs with or without sellar involvement present with a variety of nonspecific clinical manifestations. Slowly progressive hemiparesis has been recognized as the initial and most common symptoms (12, 13), indicating the progressive lesions of the basal ganglia and internal capsule area (8). In this study, hemiparesis (67.0%) is also the most common initial complaint, and many previous studies have also reported the same finding (6, 13–15). Cognitive disturbance (24.2%) follows behind, indicating an abnormality in the basal ganglia area. Signs of increased intracranial pressure, like nausea/vomiting (22.0%) and headache (16.5%), are relatively frequent symptoms. Diabetes insipidus (19.6%) is observed and may indicate tumor involvement of the sellar area.

Magnetic resonance imaging (MRI) is highly sensitive in detecting intracranial GCTs, though it is limited in distinguishing GCTs with different pathological types (3). Brain MRI is routinely performed on all patients at the first time of evaluation. Abnormalities on MRI in patients with BGGCTs involve hemiatrophy, cystic components, mass effect, intratumoral hemorrhage, and peritumoral edema (13–15). Cerebral hemiatrophy or hemorrhagic or cystic formation is highly suggestive of BGGCTs (13). Hyperintensity in T2WI and heterogeneous enhancement seem more likely to be observed. Hemiatrophy, usually presenting as ipsilateral peduncle and hemispheric atrophy, is the most common radiologic feature.

In one study, five patients, among eight cases observed with hemiatrophy on MRI, present with hemiparesis (14), which may imply an association between hemiparesis and hemiatrophy. Atrophy of the basal ganglia, even before the development of hemiparesis, was considered the earliest and most characteristic diagnostic feature in patients with BGGCTs (16).

BGGCTs can be classified as different types by the MRI features, but there is a lack of consensus on the universal standard. In the current study, we adopt a method of categorizing BGGCTs into four distinct patterns: a subtle lesion with faint or no enhancement (type 1), a small lesion <3 cm with enhancement (type 2), type 2 combined with subependymal seeding (type 3), and a large lesion ≥3 cm (type 4), among which type 1 lesions are easily misdiagnosed with non-tumorous conditions and have a longer delay in taking a biopsy (6).

Delayed diagnosis, which is defined as an interval of ≥6 months (from the onset of symptoms to the date of diagnostic MRI) (5), has been a topic of great concern in intracranial GCTs. It was considered that a longer diagnostic delay increased the risk of disseminated disease, negatively influenced treatment outcomes, left the occult lesions with enough time to develop into a full-blown disease, and significantly shorten OS (5, 6, 17). Compared with GCTs in other regions, GCTs in basal ganglia had a longer delay in diagnosis (15). In this study, delayed diagnosis was identified as a significant independent prognostic factor (OR 2.33, p = 0.045) for BGGCTs with or without sellar involvement, consistent with previous studies (6, 17). Besides, we observed that patients with BGGCTs and delayed diagnosis were more likely presenting with cognitive disturbance, mental disturbance, and diabetes insipidus (**Table 1**). In terms of clinical characteristics, delayed diagnosis was more likely in patients with multifocal lesions or tumor dissemination than that with a solitary lesion (**Table 2**). Although there is no definite cause and effect between clinical features/characteristics mentioned above and delayed diagnosis, it is recommended to carry out active diagnostic procedures in patients with high suspicion.

Different classification systems of intracranial GCTs were proposed based on histopathology, prognosis, and immunohistochemical markers. Germinoma GCT (GGCT) and NGGCTs are two main tumor patterns divided by pathological features, with the latter one further subdivided into embryonal carcinoma, endodermal sinus tumor/yolk sac tumor, choriocarcinoma, mixed GCT, and teratomas based on histology, tumor markers, and protein markers secreted by the tumor cells (18, 19). Diverse treatment outcomes and prognosis were observed in different histological subtypes, categorizing intracranial GCTs into three therapeutic groups with good, intermediate, and poor prognosis (20). Pure germinoma and mature teratoma were classified into the good prognosis group. In this study, patients with basal ganglia pure germinoma were more likely to achieve complete remission (p = 0.002) and had significantly higher OS rates and PFS rates (p = 0.003 and p = 0.001; **Figure 3**). Pure germinoma was revealed as a significant independent prognostic factor (OR 4.64, p = 0.002) for basal GCTs. Pure germinoma in basal ganglia showed similar prognostic characteristics to intracranial pure germinoma.

It was considered that distinct therapeutic modalities should be applied to fit the patients in different prognosis groups (20, 21). Germinomas, making up over half of all intracranial GCTs, are extremely sensitive to RT and CT. All patients with intracranial germinoma were recommended to receive RT (2). It was reported that more than 90% of patients with intracranial germinomas were cured with CSI, and the long-term OS was from 90% to 100% after RT alone with a total cranial dose of 40–50 Gy (22). CT was considered an effective way to reduce the dose and volume of RT without weakening the survival rates (2, 19, 21, 23, 24). CT alone was not recommended for its significantly inferior outcomes in patients with intracranial GCTs compared with CT combined with RT (21, 23, 25). Following neoadjuvant CT, WVI was necessary and sufficient for patients with localized germinoma, minimizing adverse effects of RT without affecting outcome (2, 19, 21, 22, 26). For patients with spinal dissemination, craniospinal irradiation was required. In terms of NGGCTs, multidisciplinary therapy, including tumor resection, radiation, and CT, was established (2, 19, 24, 27). Patients with BGGCTs displayed significantly worse intelligent performance and health-related quality of life than those with tumors in sellar and pineal regions (26, 28). WBI rather than WVI was suggested in patients with basal ganglia germinoma, since the WVI was likely not sufficient for the deep brain lesions (14, 28). This study observed a statistically significant difference in PFS rates (p = 0.001; **Figure 4**) between patients with basal ganglia pure germinoma receiving CSI or WVI or WBI and those receiving only PB or others. For patients with non-pure germinoma, survival analysis revealed a higher PFS rate in CSI or WVI or WBI treatment group and was not significant.

A consensus emerged that surgical biopsy was required for intracranial germ-cell tumor diagnosis without elevated AFP or HCG, regardless of radiological imaging findings (2). Despite many disadvantages, the surgical biopsy was critical to obtain an accurate pathological subtype, which predicted treatment regimens and prognosis (29, 30). The extent of surgical resection for BGGCTs is still unproven. As the evolution of neurosurgical techniques and the reduction of surgery-related morbidities, benefits may be received from more aggressive resection (14, 23). In this study, we investigated the impact of surgical debulking on clinical outcomes compared with surgical biopsy. The survival analysis of the relationship between operation types and survival was shown in **Figure 5**. Although without statistical significance, a trend was observed that the PFS rate of surgical debulking was higher than surgical biopsy in patients with basal ganglia pure germinoma ≥3 cm (p = 0.436; **Figure 5**). However, in terms of tumor size <3 cm, the surgical biopsy revealed a trend to have a better prognosis (p = 0.137; **Figure 5**). The result suggested that surgical debulking for basal ganglia pure germinoma with tumor size more than 30 mm may provide some benefits. Further studies with a large sample size are needed to check the hypothesis on surgical debulking.

There are several limitations in our study. This study is a retrospective study whose data were retrieved from electronic medical records in our institution and collected from previously published studies. Consequently, selection bias, missing data, and inaccurate information are inevitable. Patients included in this study were heterogeneous, leading to selection bias and additional confounders. For example, a higher proportion of patients in our institute presented with a combined sellar region involved, but the stratified analysis was not performed because of the small sample size. Moreover, analysis related to complications was limited in this study as a lack of adequate clinical and prognostic information. Finally, the small sample size of this study was not sufficient to give more reliable and precisive results, suggesting more patients involved in future studies.

## Conclusions

In conclusion, we discussed the clinical presentations, MRI features, delayed diagnosis, prognosis, and treatment of BGGCTs with or without sellar involvement. Young-age onset and male predominance are two outstanding features. Atrophy of the basal ganglia and hemiatrophy on MRI act as diagnostic characteristics in patients with BGGCTs, responsible for hemiparesis and cognitive disturbance. Delayed diagnosis, RT field (CSI or WVI or WBI), and pure germinoma were identified as significant independent prognostic factors. Pure germinoma in basal ganglia shows similar prognostic features to pure intracranial germinoma. RT keeps playing a pivotal role. The extent of surgical resection for BGGCTs is not yet clear. This study finds potential benefits from surgical debulking for basal ganglia pure germinoma ≥3 cm. Further studies with a large sample size are suggested to check the hypothesis on surgical debulking.

## Data Availability Statement

The raw data supporting the conclusions of this article will be made available by the authors without undue reservation.

## Ethics Statement

The studies involving human participants were reviewed and approved by the Ethics Committee of Peking Union Medical College Hospital. Written informed consent to participate in this study was provided by the participants’ legal guardian/next of kin.

## Author Contributions

Conceptualization, HZ and YY. Data curation, YZ. Formal analysis, YZ, LW. Funding acquisition, YY and YZ. Writing—original draft, YZ and LW. Writing—review and editing, LW, WM, HP, and RW. All authors contributed to the article and approved the submitted version.

## Funding

This study was supported by the Chinese Academy of Medical Sciences Innovation Fund for Medical Sciences (No. 2016-I2M-1-002) for YY and Youth Science Foundation of Peking Union Medical College Hospital (No. pumch201911867) for YZ.

## Conflict of Interest

The authors declare that the research was conducted in the absence of any commercial or financial relationships that could be construed as a potential conflict of interest.

## Publisher’s Note

All claims expressed in this article are solely those of the authors and do not necessarily represent those of their affiliated organizations, or those of the publisher, the editors and the reviewers. Any product that may be evaluated in this article, or claim that may be made by its manufacturer, is not guaranteed or endorsed by the publisher.
